# Survival Prediction in Patients Treated Surgically for Metastases of the Appendicular Skeleton—An External Validation of 2013-SPRING Model

**DOI:** 10.3390/cancers14143521

**Published:** 2022-07-20

**Authors:** Maria Anna Smolle, Ewald Musser, Marko Bergovec, Joerg Friesenbichler, Christine Linda Wibmer, Lukas Leitner, Michala Skovlund Sørensen, Michael Mørk Petersen, Iva Brcic, Joanna Szkandera, Susanne Scheipl, Andreas Leithner

**Affiliations:** 1 Department of Orthopaedics and Trauma, Medical University of Graz, 8036 Graz, Austria; maria.smolle@medunigraz.at (M.A.S.); ewald.musser@medunigraz.at (E.M.); marko.bergovec@medunigraz.at (M.B.); joerg.friesenbichler@medunigraz.at (J.F.); christine.wibmer@orthopaedie-kuerzl.at (C.L.W.); lukas.leitner@medunigraz.at (L.L.); andreas.leithner@medunigraz.at (A.L.); 2 Department of Orthopaedic Surgery, Rigshospitalet, University Hospital of Copenhagen, 2100 Copenhagen, Denmark; michala.skovlund@gmail.com; 3 Department of Clinical Medicine, University Hospital of Copenhagen, 2100 Copenhagen, Denmark; michael.moerk.petersen@regionh.dk; 4 D&R Institute of Pathology, Medical University of Graz, 8010 Graz, Austria; iva.brcic@medunigraz.at; 5 Division of Clinical Oncology, Department of Medicine, Medical University of Graz, 8036 Graz, Austria; joanna.szkandera@medunigraz.at

**Keywords:** bone metastasis, survival prediction, prognosis, orthopaedic oncology

## Abstract

**Simple Summary:**

Bone tumour metastases are highly prevalent among cancer patients. In case these have to be treated surgically due to impending or pathological fracture, estimation of patients’ life expectancy is of importance in order to choose the best treatment option possible. In the current study, the 2013-SPRING model, developed to predict prognosis of surgically treated bone metastasis patients, was validated in an external patient cohort of 303 bone metastasis patients. AUC ROCs at all three endpoints assessed (i.e., survival at 3, 6 and 12 months following surgery for bone metastases) were all above 0.780. Furthermore, stratification into type of surgery (endoprosthesis (*n* = 162) vs. osteosynthesis (*n* = 141) and metastasis location (upper limb (*n* = 65) vs. lower limb (*n* = 238)) revealed a comparable predictive accuracy of the 2013-SPRING model, albeit slightly better performance in the osteosynthesis as compared with endoprosthesis subgroup, as well as upper limb in comparison to lower limb subgroup was observed.

**Abstract:**

Introduction: The aim of this study was to externally validate the 2013-SPRING model, a survival prediction tool for patients treated surgically for bone metastases in a retrospective patient cohort from a single institution. Moreover, subgroup analyses on patients treated with (A) endoprostheses or (B) osteosynthesis, as well as (C) upper limb and (D) lower limb metastases, were performed. Methods: Altogether, 303 cancer patients (mean age: 67.6 ± 11.1 years; 140 males (46.2%)) with bone metastases to the extremities, treated surgically between March 2000 and June 2018 at a single tertiary sarcoma centre, were retrospectively included. Median follow-up amounted to 6.3 (interquartile range (IQR): 2.3–21.8) months, with all patients followed-up for at least one year or until death. The 2013-SPRING model was applied to assess the prognostication accuracy at 3, 6 and 12 months. Models were validated with area under the curve receiver operator characteristic (AUC ROC; the higher the better), as well as Brier score. Results: Of the 303 patients, 141 had been treated with osteosynthesis (46.5%), and the remaining 162 patients with endoprosthesis (53.5%). Sixty-five (21.5%) metastases were located in the upper limbs, and two hundred and thirty-eight (78.5%) in the lower limbs. Using the 2013-SPRING model for the entire cohort, the accuracy of risk of death prediction at 3, 6 and 12 months, determined by the AUC ROC, was 0.782 (95% CI: 0.729–0.843), 0.810 (95% CI: 0.763–0.858) and 0.802 (95% CI: 0.751–0.854), respectively. Corresponding Brier scores were 0.170, 0.178 and 0.169 at 3, 6 and 12 months. In the subgroup analyses, predictive accuracy of the 2013-SPRING model was likewise encouraging, albeit being slightly higher in the osteosynthesis subgroup as compared with the endoprosthesis subgroup, and also higher in the upper limb in comparison to the lower limb metastasis subgroup. Conclusions: The current validation study of the 2013-SPRING model shows that this model is clinically relevant to use in an external cohort, also after stratification for surgical procedure and metastasis location.

## 1. Introduction

A major reason for cancer-associated death is the malignant tumours’ ability to disseminate to other organs [1]. With novel antitumour therapeutics, life expectancy of cancer patients has further improved, and the number of patients living with metastatic cancer, including bones, has increased [2]. Breast, prostate, renal cell and lung cancer as well as multiple myeloma frequently give metastases to bones, eventually causing pathological fractures [3,4].

Pain caused by bone metastases, impending fractures and pathological fractures are major limitations to patients’ quality of life [5], wherefore careful, multidisciplinary management is warranted [4]. Once a tumour has metastasised, treatment is usually palliative, i.e., focusing on prevention of disease progression, alleviation of symptoms and maintenance of mobility as well as quality of life [4]. Notably, in selected tumour entities as renal cell carcinoma, singular metastases—and sometimes even oligometastases—are nowadays considered as potentially curable and treated similar to primary tumours of bone [6].

Systemic antitumour agents, bone-targeted substances as denosumab and local radiotherapy constitute the foundation of therapy in patients with bone metastases [4,7,8]. Should pain be the major symptom in absence of fracture risk, palliative radiotherapy can prove beneficial [7,9]. However, the prolonged life expectancy also increases the risk for local tumour progression and thus ensuing impending or pathological fracture.

In these cases, the surgical treatment plan has to consider patients’ prognosis, general condition, rigidity and durability of fixation chosen, as well as anticipated rehabilitation time, which should not exceed life expectancy [10]. In order to estimate prognosis of bone metastasis patients with impending or pathological fractures prior to surgery, some prognostic models have been developed in the past. These include the (modified) Bauer Score for spinal lesions [11,12], the SORG Machine-Learning Algorithm (SORG-MLA) [13] and OPTIModel [14] for both spinal and extremity metastases, as well as the PATHFx model [15], the 2008-SPRING model [16] and its updated version, the 2013-SPRING model for extremity lesions [17]. The latter model uses readily available clinical variables in order to estimate patients’ survival probability within 3, 6 and 12 months after surgery. However, it was built and validated on patient cohorts undergoing (tumour) endoprosthetic reconstruction only, whilst patients treated with osteosynthesis were not considered.

Therefore, the aim of the current retrospective study was to externally validate the 2013-SPRING model using a single-centre cohort of bone metastasis patients treated both with (tumour) endoprostheses and osteosynthesis for impending or pathological long bone fractures. Furthermore, a separate validation for upper vs. lower limb metastases was performed.

## 2. Materials and Methods

**Figure 1:**
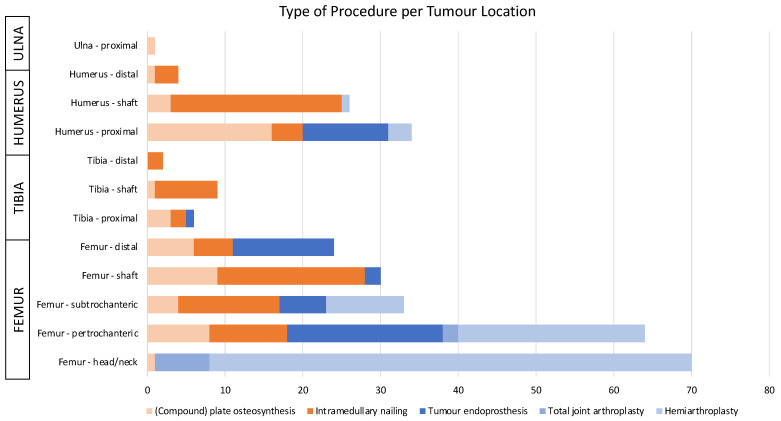
Type of surgical procedure performed separated by location of metastases within the bone.

All patients with bone metastases of the appendicular skeleton treated surgically at a single tertiary sarcoma centre between January 2000 and June 2018 were potentially eligible. Minimum follow-up was set at 12 months, or until patient death. Of initially 316 patients, 13 had to be excluded due to partially missing information or follow-up less than 12 months, resulting in 303 patients finally eligible. Mean patient age at time of surgery was 67.6 ± 11.1 years, and 140 were males (46.2%). One hundred and forty-one patients (46.5%) had undergone osteosynthesis, whilst the remaining one hundred and sixty-two patients (53.5%) had been treated with (tumour) endoprostheses (Table 1, Figure 1). The majority of metastases was located in the lower limbs (78.5%, Table 1).

All patients were followed up until last contact or visit at the local health care system, or until death. Median follow-up amounted to 6.3 (IQR: 2.3–21.8] months. At final follow-up, 276 patients had died of disease (91.1%).

### 2.1. The 2013-SPRING Model

The 2013-SPRING model as published by Sørensen et al. in 2018 was validated using the present patient cohort. This model predicts patient survival at 3, 6 and 12 months following surgery for bone metastases using logistic regression analyses for the respective endpoints [17]. Notably, the 2013-SPRING model, as well as its predecessor 2008-SPRING model [16], have been developed on cohorts of patients undergoing endoprosthetic reconstruction for impending or pathological fractures only, whilst those patients treated with, e.g., intramedullary nailing or compound plate osteosynthesis, were not included [17].

Variables required for calculation of the 2013-SPRING model were obtained from patients, i.e., underlying cancer type (grouped into slow, moderate and fast growing type, as proposed by the model’s developers [17]), haemoglobin levels two weeks to one day prior to surgery, impending or pathological fracture, presence of visceral metastases, presence of singular or multiple bone metastases (obtained on preoperative scans or scans performed up to three months postoperatively), Karnofski score (grouped in <70 or ≥70 points) and *American Society of Anesthesiologists* (ASA) score (grouped into ASA 1 and 2 vs. ASA 3 and 4). Furthermore, information on location of bone metastases and type of surgery performed was obtained.

### 2.2. Statistical Analysis

Means and medians are provided with corresponding standard deviations (SDs) and interquartile ranges (IQRs), respectively. Multivariate logistic regression analyses with endpoints 1) survival status at 3, 6 and 12 months were calculated using the proposed variables of the 2013-SPRING model. These models were validated on the entire cohort (*n* = 303) as well as separately for (A) patients treated with endoprostheses (*n* = 162) or (B) other surgical procedures (*n* = 141) and (C) patients with metastases to the upper limbs (*n* = 65) or (D) to the lower limbs (*n* = 238).

Baseline differences between surgery groups (i.e., osteosynthesis vs. endoprosthesis) and bone metastasis location (upper vs. lower limb) were assessed with chi-squared tests for binary and categorical variables, as well as t-tests for normally distributed continuous variables. For evaluation of the model’s accuracy in prediction of patient death for the three time points, Brier score and area under the curve receiver operator characteristic (AUC ROC; with 95% confidence intervals (95% CIs]) were subsequently calculated. For the Brier score (between 0 and 1), lower scores indicate higher accuracy. For AUC ROC (also between 0 and 1), higher values signify better prediction. Furthermore, positive predictive values (PPVs) and negative predictive values (NPVs) at the respective time points were calculated for the entire cohort as well as subgroups. A *p*-value of <0.05 was considered statistically significant.

## 3. Results

The most common bone metastasis location was the femur in 221 cases (72.9%), followed by the humerus in 64 cases (21.1%; Table 2). Breast cancer was the most frequent histological subtype in 77 patients (25.4%), followed by lung cancer in 68 (22.4%) and renal cell carcinoma in 55 patients (18.2%). Of the 162 patients treated with endoprostheses, the majority had undergone hemiarthroplasty (*n* = 100; 61.7%) of proximal hip or humerus, whereas 53 had received a tumour endoprosthesis (32.7%; Table 1).

### 3.1. Differences between Osteosynthesis Group (n = 141) and Endoprosthesis Group (n = 162)

There were some significant differences between patients treated with endoprostheses in comparison to those undergoing osteosynthesis, justifying the approach of additionally validating the 2013-SPRING model on the two subgroups separately. Differences included a higher proportion of patients with visceral metastases (55.4% vs. 43.2%; *p* = 0.035), a male predominance (54.9% vs. 39.5%; *p* = 0.012) and higher haemoglobin levels (7.5 ± 1.2 mM vs. 7.2 ± 1.0 mM; *p* = 0.015; Table 2) in the osteosynthesis group as compared with the endoprosthesis group. Furthermore, a significant difference in anatomical location of bone metastases with regards to surgical type was present, with most endoprosthetic reconstructions being performed in metastases to the femur (90.1% vs. 53.2%; *p* < 0.001). Moreover, a higher proportion of pathological fractures than impending fractures was treated with (tumour) endoprostheses (74.7% vs. 63.1%; *p* = 0.029; Table 2).

### 3.2. Differences between Upper Limb (n = 65) and Lower Limb (n = 238)

Between patients with upper limb and lower limb metastases, some differences were likewise found, wherefore the 2013-SPRING model was also validated in this subgroup. Patients with upper limb metastases were significantly more likely to undergo osteosynthesis (*n* = 50; 76.9%) than those with metastases to the lower limbs (*n* = 91; 38.2%; *p* < 0.001). However, the proportion of intramedullary nailing and (compound) osteosyntheses was comparable (upper vs. lower limb—58.0% (*n* = 29) vs. 64.8% (*n* = 59) intramedullary nailing; *p* = 0.423). Furthermore, patients with metastases of the upper limb significantly more often presented with pathological fractures (*n* = 54; 83.1%) than patients with metastases to the lower limbs (*n* = 156; 65.6%; *p* = 0.007; Table 2).

With regards to haemoglobin levels (*p* = 0.058), presence of multiple bone metastases (*p* = 0.428) or visceral metastases (*p* = 0.294), Karnofski score ≥70 (*p* = 0.176), ASA (*p* = 0.087), primary cancer growth (*p* = 0.573), patient age (*p* = 0.090) or gender (*p* = 0.163), no significant differences were found (Table 2).

### 3.3. Validation of 3-, 6- and 12-Month Risk of Death

**Figure 2:**
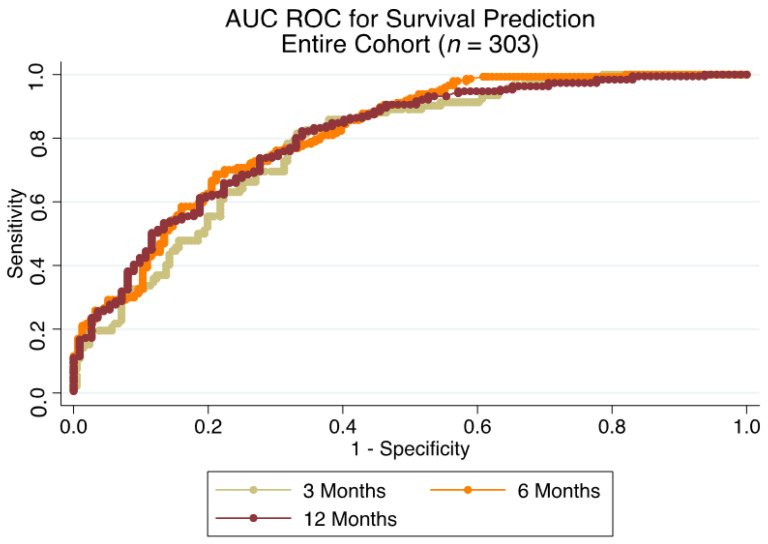
Accuracy of survival prediction for all patients treated surgically (*n* = 303) for bone metastases of the appendicular skeleton at 3, 6 and 12 months with receiver operator characteristic area under the curve (ROC AUC), based on the 2013-SPRING model.

For the entire cohort (*n* = 303), AUC ROC for risk of death prediction at 3, 6 and 12 months was 0.782 (95% CI: 0.729–0.843), 0.810 (95% CI: 0.763–0.858) and 0.802 (95% CI: 0.751–0.854), respectively (Figure 2). Corresponding Brier score amounted to 0.170, 0.178 and 0.169 at 3, 6 and 12 months. PPVs and NPVs at the respective time points are listed in Table 3.

#### 3.3.1. Subgroup Analysis—Osteosynthesis Group (*n* = 141) vs. Endoprosthesis Group (*n* = 162)

**Figure 3:**
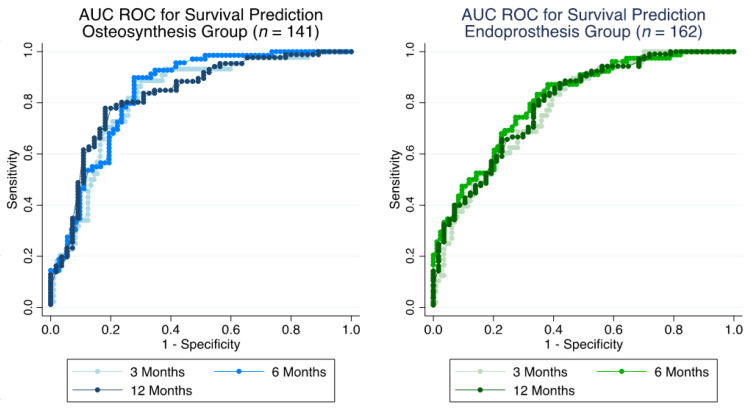
Accuracy of survival prediction for patients treated with osteosynthesis (*n* = 141, (**left**)) and endoprostheses (*n* = 162, (**right**)) for bone metastases of the appendicular skeleton at 3, 6 and 12 months with receiver operator characteristic area under the curve (ROC AUC), based on the 2013-SPRING model.

For the osteosynthesis group only, AUC ROC was 0.821 (95% CI: 0.749–0.893) at 3 months, 0.841 (95% CI: 0.774–0.907) at 6 months and 0.829 (95% CI: 0.756–0.901) at 12 months (Figure 3). Accuracy as estimated with Brier score was 0.160 at 3 months, 0.158 at 6 months and 0.184 at 12 months.

Risk of death prediction for the endoprosthesis group revealed AUC ROC of 0.782 (95% CI: 0.709–0.854) at 3 months, 0.809 (95% CI: 0.744–0.874) at 6 months and 0.794 (95% CI: 0.721–0.867) at 12 months (Figure 3). Brier score amounted to 0.167, 0.181 and 0.169 at 3, 6 and 12 months, respectively.

PPVs and NPVs at the respective time points are listed in Table 3.

#### 3.3.2. Subgroup Analysis—Upper Limb (*n* = 65) vs. Lower Limb (*n* = 238)

**Figure 4:**
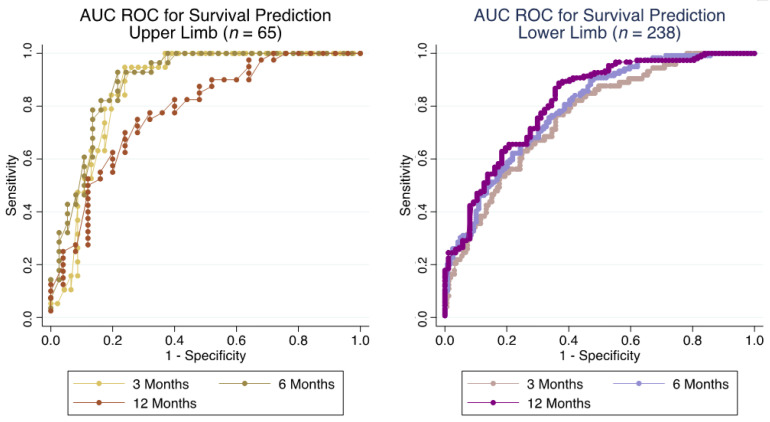
Accuracy of survival prediction for patients with upper limb metastases (*n* = 65, (**left**)) and lower limb metastases (*n* = 238, (**right**)) for bone metastases of the appendicular skeleton at 3, 6 and 12 months with receiver operator characteristic area under the curve (ROC AUC), based on the 2013-SPRING model.

For metastases located in the upper limbs, risk of death prediction showed AUC ROCs of 0.866 (95% CI: 0.778–0.953), 0.893 (95% CI: 0.815–0.971) and 0.781 (95% CI: 0.662–0.900), at 3, 6 and 12 months (Figure 4). Brier scores at the respective time points were 0.138, 0.130 and 0.178.

For lower limb metastases, risk of death prediction revealed AUC ROCs of 0.762 (95% CI: 0.699–0.825) at 3 months, AUC ROCs of 0.792 (95% CI: 0.736–0.848) at 6 months and AUC ROCs of 0.814 (95% CI: 0.757–0.871) at 12 months (Figure 4). Corresponding Brier scores were 0.175, 0.186 and 0.162 at 3, 6 and 12 months, respectively.

PPVs and NPVs at the three time points are visible in Table 3.

## 4. Discussion

In the current study, the external validation of the 2013-SPRING model revealed a promising survival prediction at 3, 6 and 12 months after surgery for metastatic bone disease in an independent cohort of patients with bone metastases to the appendicular skeleton. Additionally, subgroup analysis for patients treated by endoprostheses (as in the original model) and osteosynthesis, as well as for patients with metastases to the upper limb and lower limb, revealed comparable survival prediction accuracy depending on treatment group and metastasis location.

Survival prediction in bone metastasis patients with impending or pathological fractures is necessary in order to select the most suitable treatment option. The utmost goal is quality of life-maintenance and -improvement, wherefore a stable situation with early mobilisation, full weight bearing and short in-hospital stay must be strived for [4].

However, patients may outlive complications associated with one or the other treatment option, especially in case metastatic bone is not (partially) removed during surgery as upon intramedullary stabilisation. Consequently, members of the Musculoskeletal Tumour Society (MSTS) agreed that life expectancy beyond 6 months in bone metastasis patients with impending or pathological fractures would justify the use of more durable implants [18]. Although indications for different surgical procedures (i.e., intramedullary nailing, (compound) plate osteosynthesis, hemiarthroplasty, tumour endoprosthesis) in the current study are in line with literature recommendations [10], these are mainly based on experience and retrospective analyses [10], whilst prospective studies investigating the most suitable surgical approach are yet to be performed [19].

In order to aid decision making, some models to predict survival in bone metastasis patients with impending or pathological fractures have been developed in the past [13,15,16], including the 2013-SPRING model [17]. Their validation—both internally and externally—is necessary in order to evaluate whether models tend to over- or underestimate remaining prognosis [20].

Herein, validation of the 2013-SPRING model using the entire patient cohort revealed a reliable predictive accuracy, with AUC ROCs > 0.780 at all three end points. Notably, in the original study by Sørensen et al. [17], higher AUC ROCs than in the present study were found, all exceeding 0.820. However, the authors only included patients treated with endoprostheses to develop the model. Thus, separate analyses on patients treated with endoprostheses or osteosynthesis were herein performed.

At baseline, some differences in variables necessary to construct the 2013-SPRING model were found depending on treatment group; patients receiving endoprostheses were rather female, more likely to have metastases in the femur, present with pathological fractures and lower haemoglobin levels, but were less likely to have visceral metastases.

Despite these marked differences and differing surgical philosophies, discrepancies in predictive accuracy were less pronounced. Interestingly, subgroup analysis on patients treated with osteosynthesis revealed even slightly better AUC ROCs and Brier scores at all three endpoints than the analysis on the endoprosthesis subgroup, even though the original model had been developed on patients treated with endoprostheses. The retrospective design, non-Scandinavian population and non-random assignment of patients to specific treatments (i.e., osteosynthesis vs. endoprosthesis) may all have contributed to this finding. Regardless of the subgroup analysed, any AUC ROC was >0.780. This is indicative of reliable survival prediction in bone metastasis patients, also in a cohort not originally considered upon model development.

Whilst one main goal of surgery for lower limb metastases is stabilisation enabling full weight bearing, this is of lesser significance in metastases to the upper limbs, as bones in this region are not primarily weight bearing [10]. Yet, torsional forces have to be considered, especially in metastases to the proximal humerus [10,21]. This may thus lead to differences in surgical techniques applied. Correspondingly, osteosyntheses were significantly more often performed in metastases to the upper limbs, whilst patients with metastases to the lower limbs were more likely to undergo endoprosthetic reconstruction. On the other hand, the proportion of (compound) plate osteosynthesis and intramedullary nailing was comparable between upper and lower limb metastases. Notably, significantly more patients with upper limb metastases already presented with pathological fractures than patients with lower limb lesions, which may be related to the fact that pain as a sign of impending fracture [22] more likely develops in weight bearing bones, leading to earlier patient referral. Nonetheless, further subgroup analysis depending on metastasis location revealed a reliable survival prediction accuracy in both lower and upper limb metastases, with the latter group even reaching slightly higher accuracy values.

Limitations of the study include the long inclusion period upon which patients were recruited, wherefore potential changes in prognosis due to refined (systemic) treatment options could not be considered. This may bias the validation results as treatment strategies have changed over the period. Moreover, as no prospective analysis had been performed, a certain impact of prognosis-adjusted treatment decisions cannot be eliminated.

Furthermore, this validation study focused on bone metastases to the appendicular skeleton only, since the 2013-SPRING model has been developed for patients with long bone metastases. In addition, we did not investigate emerging complications or additional treatments administered, as the main scope of this study was to externally validate the 2013-SPRING model regarding its risk of death prediction.

## 5. Conclusions

Surgical treatment of bone metastasis patients with impending or pathological fractures has to consider remaining life expectancy in order to select the most viable therapeutic approach. The external validation of the 2013-SPRING model using an independent cohort of patients likewise treated with endoprostheses or osteosynthesis revealed a reliable accuracy regarding survival prediction for both surgical strategies, as well as for upper vs. lower limb metastases.

## Figures and Tables

**Table 1:** Types of surgeries performed, separated by bone metastasis location.

Bone Metastasis Location	Lower Limb (*n* = 238)		Upper Limb (*n* = 65)	
	Femur (*n* = 221)	Tibia (*n* = 17)	Humerus (*n* = 64)	Ulna (*n* = 1)
**Osteosynthesis Group (*n* = 141; 100%)**				
(Compound) plate osteosynthesis (*n* = 53; 37.6%)	28	4	20	1
Intramedullary nailing (*n* = 88; 62.4%)	47	12	29	0
**Endoprosthesis Group (*n* = 162; 100%)**				
Tumour endoprosthesis (*n* = 53; 32.7%)	41	1	11	0
Total joint arthroplasty (*n* = 9; 5.6%)	9	0	0	0
Hemiarthroplasty (*n* = 100; 61.7%)	96	0	4	0

**Table 2:** Patient demographics, split by type of surgery (endoprosthesis vs. osteosynthesis) and metastasis location (upper vs. lower limb).

		Entire Cohort (*n* = 303)	Osteosynthesis Group (*n* = 141)	Endoprosthesis Group (*n* = 162)	*p*-Value	Upper Limb (*n* = 65)	Lower Limb (*n* = 238)	*p*-Value
**Gender**	Female	163 (53.5)	65 (46.1)	98 (60.5)	**0.012**	30 (46.2)	133 (55.9)	0.163
	Male	140 (46.5)	76 (53.9)	64 (39.5)		35 (53.8)	105 (44.1)	
**Bone Metastasis Location**	Femur	221 (72.9)	75 (53.2)	146 (90.1)	**<0.001**	N/A		
	Humerus	64 (21.1)	49 (34.8)	15 (9.3)				
	Tibia	17 (5.6)	16 (11.3)	1 (0.6)				
	Ulna	1 (0.4)	1 (0.7)	0 (0.0)				
**Age at Surgery** (in years, mean ± SD)		67.6 ± 11.1	68.1 ± 10.4	67.1 ± 11.7	0.397	69.6 ± 10.8	67.0 ± 11.2	0.090
**Primary Cancer Growth**	Slow	95 (31.4)	36 (25.5)	59 (36.4)	0.089	20 (30.8)	75 (31.5)	0.573
	Moderate	83 (27.4)	39 (27.7)	44 (27.2)		21 (32.3)	62 (26.1)	
	Fast	125 (41.2)	66 (46.8)	59 (36.4)		24 (36.9)	101 (42.4)	
**ASA**	1 + 2	45 (14.9)	26 (18.4)	19 (11.7)	0.101	14 (21.5)	31 (13.0)	0.087
	3 + 4	258 (85.1)	115 (81.6)	143 (88.3)		51 (78.5)	207 (87.0)	
**Karnofski Score**	< 70	139 (45.9)	59 (41.8)	80 (49.4)	0.189	25 (38.5)	114 (47.9)	0.176
	≥ 70	164 (54.1)	82 (58.2)	82 (50.6)		40 (61.5)	124 (52.1)	
**Visceral Metastases**	No	155 (51.2)	63 (44.7)	92 (56.8)	**0.035**	37 (56.9)	118 (49.6)	0.294
	Yes	148 (48.8)	78 (55.3)	70 (43.2)		28 (43.1)	120 (50.4)	
**Multiple Bone Metastases**	No	86 (28.4)	43 (30.5)	43 (26.5)	0.446	21 (32.3)	65 (27.3)	0.428
	Yes	217 (71.6)	98 (69.5)	119 (73.5)		44 (67.7)	173 (72.7)	
**Fracture Type**	Impending	93 (30.7)	52 (36.9)	41 (25.3)	**0.029**	11 (16.9)	82 (34.5)	**0.007**
	Pathologic	210 (69.3)	89 (63.1)	121 (74.7)		54 (83.1)	156 (65.5)	
**Haemoglobin Levels** (in mM; mean ± SD)		7.3 ± 1.1	7.5 ± 1.2	7.2 ± 1.0	**0.015**	7.6 ± 1.2	7.3 ± 1.1	0.058

**Table 3:** Positive predictive values (PPVs) and negative predictive values (NPVs) for the entire cohort (*n* = 303), as well as treatment (osteosynthesis vs. endoprosthesis) and metastasis location subgroups (upper vs. lower limb).

Time Since Surgery		3 Months		6 Months		12 Months	
		PPV	NPV	PPV	NPV	PPV	NPV
**Entire Cohort (*n* = 303)**		54.0%	75.8%	74.1%	73.2%	77.8%	71.4%
**Type of** **Surgery**	Osteosynthesis (*n* = 141)	62.5%	78.0%	73.3%	78.8%	79.1%	72.0%
	Endoprosthesis (*n* = 162)	58.3%	78.6%	72.9%	70.7%	79.0%	68.8%
**Metastasis** **Location**	Upper Limb (*n* = 65)	66.7%	85.1%	79.3%	86.1%	74.5%	72.2%
	Lower Limb (*n* = 238)	60.5%	75.9%	69.5%	69.2%	79.4%	76.5%

## Data Availability

The data of this study are available upon reasonable request from the corresponding author.
